# Risk factors and development of a predictive model for post-traumatic cognitive impairment in patients with frontal lobe cerebral contusion

**DOI:** 10.3389/fnins.2026.1921933

**Published:** 2026-08-27

**Authors:** Wei Quan, Ming Liu, Zhuohui Lai, Wenxing Hang, Libin Fan, Liang Mi, Meng Nie, Zengguang Wang, Rongcai Jiang

**Affiliations:** 1 Tianjin Medical University, Tianjin, China; 2 Department of Neurosurgery, Tianjin Medical University General Hospital, Tianjin, China; 3 Department of Neurosurgery, Xuanwu Hospital, Capital Medical University, Beijing, China

**Keywords:** anosmia, anterior skull base fracture, cognitive impairment, frontal lobe cerebral contusion, risk factor analysis

## Abstract

**Objective:**

To identify factors associated with post-traumatic cognitive impairment in patients with frontal lobe cerebral contusion and to develop a predictive model to estimate the risk of cognitive dysfunction. This study aims to facilitate early identification of high-risk patients and support the development of individualized clinical interventions.

**Methods:**

Cognitive function was assessed using the Mini-Mental State Examination (MMSE) and the Montreal Cognitive Assessment (MoCA) at 1 week, 1 month, and 6 months after injury in patients with frontal lobe cerebral contusion. Overall recovery and functional independence were evaluated using the Barthel Index (BI) and the Extended Glasgow Outcome Scale (GOSE) at 3 and 6 months post-injury. Correlation analyses were performed to explore the associations between overall cognitive performance and clinical variables, including injury severity, anterior skull base fracture, and anosmia. Potential predictors were initially screened using univariate logistic regression analysis, and variables with *P* < 0.05 were included as candidate predictors. These variables were subsequently incorporated into a Firth logistic regression model to construct a predictive model for cognitive impairment. Model discrimination was evaluated using the receiver operating characteristic (ROC) curve, while calibration was assessed using the Hosmer–Lemeshow goodness-of-fit test and a calibration curve. In addition, decision curve analysis (DCA) was performed to evaluate the clinical utility and net benefit of the model in clinical decision-making.

**Results:**

A total of 206 patients with mild-to-moderate frontal lobe cerebral contusion were included. Univariate logistic regression analysis demonstrated that age, unilateral versus bilateral injury, contusion and hemorrhage volume, presence of anterior skull base fracture, cerebrospinal fluid (CSF) rhinorrhea, anosmia during treatment, and mechanism of injury were significantly associated with the occurrence of cognitive impairment (*P* < 0.05). Multivariate logistic regression analysis revealed that age, anosmia during treatment, and mechanism of injury were independent predictors of cognitive impairment. A predictive model was subsequently developed based on these variables. ROC analysis showed that the area under the curve (AUC) for predicting cognitive impairment was 0.83 (95% CI: 0.773–0.887, *P* < 0.05). The optimal cutoff value was 0.369, yielding a sensitivity of 79.8% and a specificity of 73.5%. The calibration curve demonstrated good agreement between predicted probabilities and observed outcomes. Decision curve analysis further indicated that the model provided a favorable net clinical benefit across a range of threshold probabilities.

**Conclusion:**

Age, anosmia during treatment, and mechanism of injury are independent risk factors for cognitive impairment in patients with mild-to-moderate frontal lobe cerebral contusion. The predictive model constructed based on these factors demonstrated good discrimination and acceptable calibration, indicating potential clinical value for risk stratification and early intervention.

## Introduction

Frontal lobe cerebral contusion is one of the most common types of traumatic brain injury (TBI) (Liu et al., 2023). Because the frontal lobe plays a critical role in cognitive processing, behavioral regulation, and emotional control, injury to this region frequently results in varying degrees of cognitive impairment, including deficits in attention, memory, and executive function (Lengenfelder et al., 2015; Tandetnik et al., 2021). These impairments can significantly affect patients’ quality of life and social reintegration. With advances in the management of TBI, patient survival rates have improved substantially. However, post-traumatic cognitive impairment has increasingly become a major determinant of long-term prognosis.

In clinical practice, cognitive function is commonly evaluated using the MMSE and the MoCA, while overall functional recovery is often assessed using BI and the GOSE (Arevalo-Rodriguez et al., 2021; Nasreddine et al., 2005; Viosca et al., 2005). However, although the assessment scales can effectively evaluate cognitive function, they primarily reflect the patient’s current status and are insufficient to explain the variability in cognitive recovery among individuals. Clinical observations have shown that patients with frontal lobe contusion exhibit significant inter-individual differences in post-injury cognitive recovery, which may be influenced by multiple factors, including injury severity, anterior skull base fractures, and anosmia.

Previous studies have largely focused on single-factor analyses, while research on multifactorial predictive modeling remains relatively limited, and there is a lack of prediction models with clear clinical applicability. In recent years, the use of multivariable statistical approaches to develop predictive models has been increasingly applied in clinical prognostic assessment, providing important support for risk stratification and individualized intervention. Among these methods, Firth logistic regression, as a bias-reduction technique, demonstrates good stability and reliability, particularly in studies with small sample sizes or low event rates.

Therefore, the present study aimed to evaluate cognitive function at multiple time points following frontal lobe cerebral contusion, identify factors associated with post-traumatic cognitive impairment, and develop a predictive model using Firth logistic regression. The model was further evaluated using ROC curves, calibration analysis, and decision curve analysis, with the goal of providing a useful tool for early identification of patients at high risk of cognitive dysfunction and for guiding individualized rehabilitation strategies.

The frontal lobe is particularly vulnerable to traumatic injury because of its anatomical location adjacent to the irregular surfaces of the anterior cranial fossa. It plays a central role in executive function, attention, working memory, behavioral regulation, and goal-directed activities. Nevertheless, post-traumatic cognitive impairment does not result solely from focal frontal damage but may also reflect disruption of distributed frontal-subcortical and frontal-limbic networks. In the present study, we restricted the cohort to patients with predominant frontal lobe cerebral contusion to reduce anatomical heterogeneity and develop a clinically applicable prediction model within a relatively homogeneous TBI population. This approach was intended to improve the interpretability of the model rather than to imply that cognitive impairment following TBI is exclusively attributable to frontal lobe injury.

## Materials and methods

### Study design and participants

**FIGURE 1:**
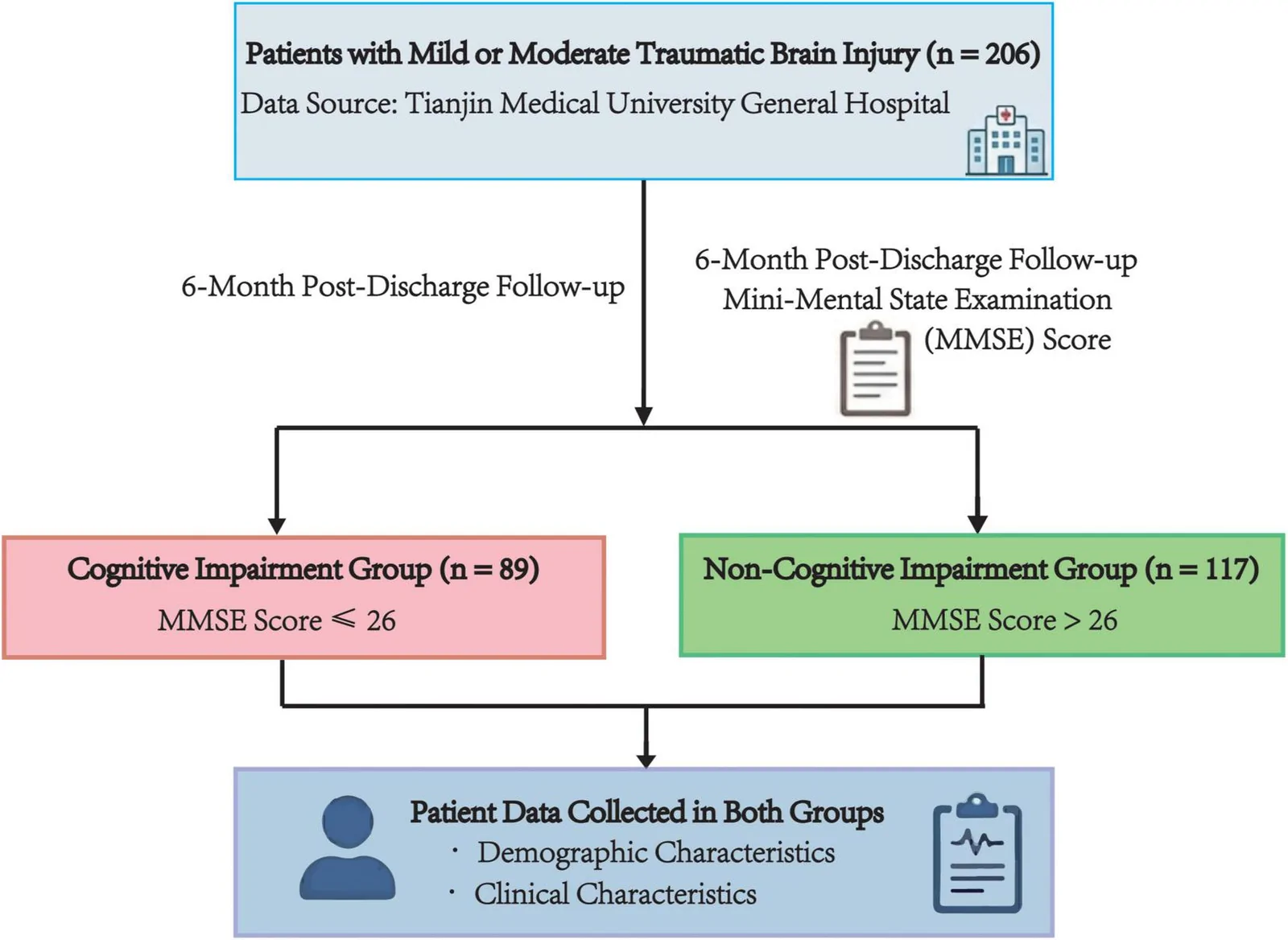
Subject selection and grouping flowchart.

This single-center observational study included 206 patients who were hospitalized for frontal lobe cerebral contusion in the Department of Neurosurgery at Tianjin Medical University General Hospital between January 2021 and June 2024. Patients were divided into a cognitive impairment group and a non-cognitive impairment group according to the presence or absence of cognitive impairment at the 6-month follow-up after discharge. The severity of was classified based on the Glasgow Coma Scale (GCS) score at admission. Patients with GCS scores of 13–15 were defined as having mild TBI, whereas those with GCS scores of 9–12 were classified as having moderate TBI. The study was conducted in accordance with the Declaration of Helsinki and approved by the Ethics Committee of Tianjin Medical University General Hospital (Approval No:IRB2026-YX-231-01). As an observational study, only routine clinical data were collected. Verbal informed consent was obtained from all patients or their family members during follow-up. The participant selection and grouping process is illustrated in Figure 1.

### Inclusion and exclusion criteria

Inclusion criteria: (1) Emergency admission with confirmed TBI, with cranial CT demonstrating frontal lobe cerebral contusion as the predominant intracranial lesion; (2) No previous history of cognitive impairment, psychiatric disorders, or TBI; (3) No prior history of neurosurgical craniotomy; (4) No use of medications affecting neurocognitive function prior to admission, such as psychotropic drugs or anticholinergic agents.

Exclusion criteria: (1) Patients initially diagnosed with mild or moderate TBI whose condition deteriorated and subsequently required decompressive craniotomy; (2) Pre-existing diseases associated with cognitive impairment, including encephalitis, brain tumors, hydrocephalus, or severe hepatic or renal dysfunction; (3) Patients with psychiatric disorders or a history of TBI prior to the current injury; (4) Use of medications affecting cognitive function before trauma; (5) History of alcohol abuse or illicit drug use that may affect cognitive function; (6) Patients with extensive cerebral contusions involving non-frontal brain regions (e.g., temporal, parietal, or occipital lobes) when these lesions were considered the predominant intracranial injury.

### Data collection

Clinical data were collected from electronic and paper medical records by trained investigators. Baseline variables recorded at admission included age, sex, GCS score at admission, injury laterality (unilateral or bilateral), past medical history (hypertension, ICH, CAD, and ischemic stroke), frontal lobe cerebral contusion and hemorrhage volume, and mechanism of injury. Mechanisms of injury included falls, e-bike accident with helmet, e-bike accident without helmet, and motor vehicle accident. Hospitalization data included length of stay (LOS), clinical complications (CSF rhinorrhea, anterior skull base fracture, EDH, SDH or SAH, anosmia, and delayed-onset cerebral contusion and hemorrhage), duration of mannitol therapy and average daily dose of mannitol, and GCS score on discharge day.

### Follow-up and outcome measures

#### Follow-up

Follow-up assessments were conducted by trained investigators through standardized telephone interviews. Patients were evaluated and relevant data were collected at different time points after discharge. At 1 week after discharge, the MMSE score, MoCA score, and GCS score were recorded. At 1 month after discharge, the MMSE score, MoCA score, and BI were recorded. At 6 months after discharge, the MMSE score, MoCA score, BI, and GOSE were recorded.

#### Outcome measures

The primary outcome of this study was the occurrence of cognitive impairment after frontal lobe cerebral contusion. Cognitive function was assessed using the MMSE and the MoCA at 1 week, 1 month, and 6 months after discharge. Patients were categorized into two groups according to their MMSE scores: Cognitive impairment group: MMSE ≤ 26; Non–cognitive impairment group: MMSE > 26. The secondary outcomes included functional recovery and neurological prognosis. Functional independence in activities of daily living was evaluated using the BI at 1 month and 6 months after discharge. Overall neurological outcome was assessed using the GOSE at 6 months after discharge.

### Statistical analysis

First, cognitive function and overall functional recovery at different time points after injury were evaluated. Correlation analyses were performed to explore the associations between cognitive function and clinical variables such as injury severity, anterior skull base fracture, and anosmia.

Second, univariate logistic regression analysis was performed to identify potential predictors of cognitive impairment. Variables with *P* < 0.05 were included as candidate predictors and subsequently entered into a Firth logistic regression model for multivariate analysis to construct a predictive model.

Finally, the performance of the predictive model was evaluated. Model discrimination was assessed using the receiver operating characteristic (ROC) curve and the area under the curve (AUC). Model calibration was evaluated using the Hosmer–Lemeshow goodness-of-fit test and calibration curves. Decision curve analysis (DCA) was performed to assess the clinical net benefit of the model across different threshold probabilities.

All statistical analyses were performed using SPSS version 27 (IBM Corp., Armonk, NY, United States) and R version 4.5.1 (R Foundation for Statistical Computing, Vienna, Austria). A two-sided *P* < 0.05 was considered statistically significant.

## Results

### Study population and baseline characteristics

A total of 206 patients with frontal lobe cerebral contusion were included in this study. Among them, 103 were male (50.0%) and 103 were female (50.0%), with a mean age of 58.6 ± 6.7 years. According to injury severity, 98 patients (50.0%) had mild TBI and 98 patients (50.0%) had moderate TBI. Anterior skull base fractures were present in 61 patients (29.6%), and 20 patients (9.7%) exhibited anosmia. During follow-up, 89 patients developed varying degrees of cognitive impairment at 6 months after injury, accounting for 43.2% of the total sample. The baseline demographic and clinical characteristics of the study population are summarized in Table 1.

**TABLE 1:** Baseline characteristics of the study population (*N* = 206).

Variable	Total (*N* = 206)
Demographic characteristics	
Age, years	58.6 ± 6.7
Sex, n (%)	
Male	103(50.0)
Female	103(50.0)
Mechanism of injury, n (%)	
Fall	103(50.0)
E-bike accident with helmet	3(1.5)
E-bike accident without helmet	83(40.3)
Motor vehicle accident	17(8.3)
Clinical characteristics	
Admission GCS (points)	13(4)
Severity of traumatic brain injury, n (%)	
mTBI	98(50.0)
moderate TBI	98(50.0)
Injury laterality, n (%)	
Unilateral	121(58.7)
Bilateral	85(41.3)
Contusion and hemorrhage volume	14.1 ± 3.6
Concomitant skull fracture, n (%)	61(29.6)
Concomitant EDH, n (%)	34(16.5)
Concomitant SDH and SAH, n (%)	118(57.3)
Concomitant CSF rhinorrhea, n (%)	22(10.7)
History of hypertension, n (%)	66(32.0)
History of ICH, n (%)	9(4.4)
History of ischemic stroke, n (%)	28(13.6)
History of CAD, n (%)	38(18.4)
Anosmia, n (%)	20(9.7)
Duration of mannitol therapy	13(5)
Average daily dose of mannitol	468.8(35.7)
Delayed-onset cerebral contusion and hemorrhage, n (%)	28(13.6)
Length of Hospital Stay	15(6)
GCS on discharge day	15(1)
GCS score at 1 week post-discharge	14(3)
MMSE score at 1 week post-discharge	23(3)
MoCA score at 1 week post-discharge	22(3)
MMSE score at 1 month post-discharge	25(2)
MoCA score at 1 month post-discharge	25(2.8)
Barthel index at 1 month post-discharge	95(5)
MMSE score at 6 months post-discharge	27(4)
MoCA score at 6 months post-discharge	28(4)
Barthel index at 6 months post-discharge	100(5)
GOS-E score at 6 months post-discharge	8(1)
Cognitive dysfunction	89(43.2)

CAD, coronary artery disease; CSF, Cerebrospinal Fluid; EDH, Epidural Hematoma; ICH, intracerebral hemorrhage; SDH, Subdural Hematoma; SAH, Subarachnoid Hemorrhage; mTBI, mild Traumatic Brain Injury; GCS, Glasgow Coma Scale; MMSE, Mini-Mental State Examination; MoCA, Montreal Cognitive Assessment; GOS-E, Glasgow Outcome Scale Extended. Data are presented as mean ± standard deviation (SD) for normally distributed variables, median (interquartile range, IQR) for non-normally distributed variables, and number (percentage) for categorical variables.

### Comparison of clinical characteristics between the cognitive impairment and non-cognitive impairment groups

Comparisons of clinical characteristics between the two groups are presented in Table 2.

**TABLE 2:** Comparison between patients with and without cognitive impairment.

Variable	Cognitive dysfunction group (*n* = 89)	Non-cognitive dysfunction group (*n* = 117)	*P*-value
Demographic characteristics			
Age, years	62.13 ± 6.06	55.89 ± 5.85	< 0.001
Sex, n (%)			
Male	46 (51.7)	57 (48.7)	0.778
Female	43 (48.3)	60 (51.3)	0.778
Mechanism of injury, n (%)			
Fall	28 (31.5)	75 (64.1)	< 0.0001
E-bike accident with helmet	3 (3.4)	0 (0.0)	< 0.0001
E-bike accident without helmet	45 (50.6)	38 (32.5)	< 0.0001
Motor vehicle accident	13 (14.6%)	4 (3.4%)	< 0.0001
Clinical characteristics			
Admission GCS (points)	13(5)	13(4)	0.895
Severity of traumatic brain injury, n (%)			
mTBI	46 (51.7)	62 (53.0)	0.964
moderate TBI	43 (48.3)	55 (47.0)	0.964
Injury laterality, n (%)			
Unilateral	45 (50.6)	76 (65.0)	0.053
Bilateral	44 (49.4)	41 (35.0)	0.053
Frontal lobe cerebral contusion and hemorrhage volume	14.90 ± 4.04	13.50 ± 3.18	0.006
Concomitant anterior skull base fracture, n (%)	39 (43.8)	22 (18.8)	< 0.001
Concomitant EDH, n (%)	18 (20.2)	16 (13.7)	0.287
Concomitant SDH and SAH, n (%)	55 (61.8)	63 (53.8)	0.317
Concomitant CSF rhinorrhea, n (%)	14 (15.7)	8 (6.8)	0.069
History of hypertension, n (%)	33 (37.1)	33 (28.2)	0.230
History of ICH, n (%)	4 (4.5)	5 (4.3)	1.000
History of ischemic stroke, n (%)	12 (13.5)	16 (13.7)	1.000
History of CAD, n (%)	17 (19.1)	21 (17.9)	0.976
Anosmia, n (%)	16 (18.0)	4 (3.4)	0.001
Duration of mannitol therapy	13(5)	13(5)	0.371
Average daily dose of mannitol	468.75(35.72)	468.75(35.72)	0.364
Delayed-onset cerebral contusion and hemorrhage, n (%)	14 (15.7)	14 (12.0)	0.565
Length of Hospital Stay	16(8)	15(6)	0.186
GCS on discharge day	15(1)	15(1)	0.593
GCS score at 1 week post-discharge	13(4)	14(3)	< 0.0001
MMSE score at 1 week post-discharge	22(2)	23(3)	< 0.0001
MoCA score at 1 week post-discharge	21(2)	23(3)	0.014
MMSE score at 1 month post-discharge	24(3)	25(2)	< 0.0001
MoCA score at 1 month post-discharge	24(3)	26(2)	< 0.0001
BI at 1 month post-discharge	95(5)	100(5)	< 0.0001
MMSE score at 6 months post-discharge	25(1)	9(1)	< 0.0001
MoCA score at 6 months post-discharge	24(1)	29(1)	< 0.0001
BI at 6 months post-discharge	95(5)	100(5)	< 0.0001
GOS-E score at 6 months post-discharge	7(1)	8(1)	< 0.0001

BI, Barthel Index; CAD, coronary artery disease; CSF, Cerebrospinal Fluid; EDH, Epidural Hematoma; ICH, intracerebral hemorrhage; SDH, Subdural Hematoma; SAH, Subarachnoid Hemorrhage; mTBI, mild Traumatic Brain Injury; GCS, Glasgow Coma Scale; MMSE, Mini-Mental State Examination; MoCA, Montreal Cognitive Assessment; GOS-E, Glasgow Outcome Scale Extended. Continuous variables were compared using Student’s *t*-test or the Mann–Whitney U test, as appropriate. Categorical variables were compared using the chi-square test or Fisher’s exact test. A two-tailed *P* < 0.05 was considered statistically significant.

In terms of demographic characteristics, patients in the cognitive impairment group were significantly older than those in the non-cognitive impairment group (62.13 ± 6.06 vs. 55.89 ± 5.85 years, *P* < 0.001). However, no significant difference in sex distribution was observed between the two groups (*P* = 0.778). Regarding mechanisms of injury, the distribution differed significantly between the two groups (*P* < 0.0001). In particular, e-bike accident without helmet were more common in the cognitive impairment group. In terms of injury severity, patients in the cognitive impairment group had significantly lower GCS scores at admission than those in the non–cognitive impairment group (*P* < 0.001). In addition, the volume of cerebral contusion and hemorrhage was greater in the cognitive impairment group. Imaging findings showed that the incidence of anterior skull base fractures was significantly higher in the cognitive impairment group than in the non–cognitive impairment group (43.8% vs. 18.8%, *P* = 0.006). Notably, the incidence of anosmia was also significantly higher in the cognitive impairment group (18.0% vs. 3.4%, *P* = 0.001). During follow-up, patients in the cognitive impairment group had significantly lower scores on the MMSE, MoCA, BI and GOSE compared with those in the non–cognitive impairment group (all P < 0.001) (Table 2).

### Univariate logistic regression analysis

**FIGURE 2:**
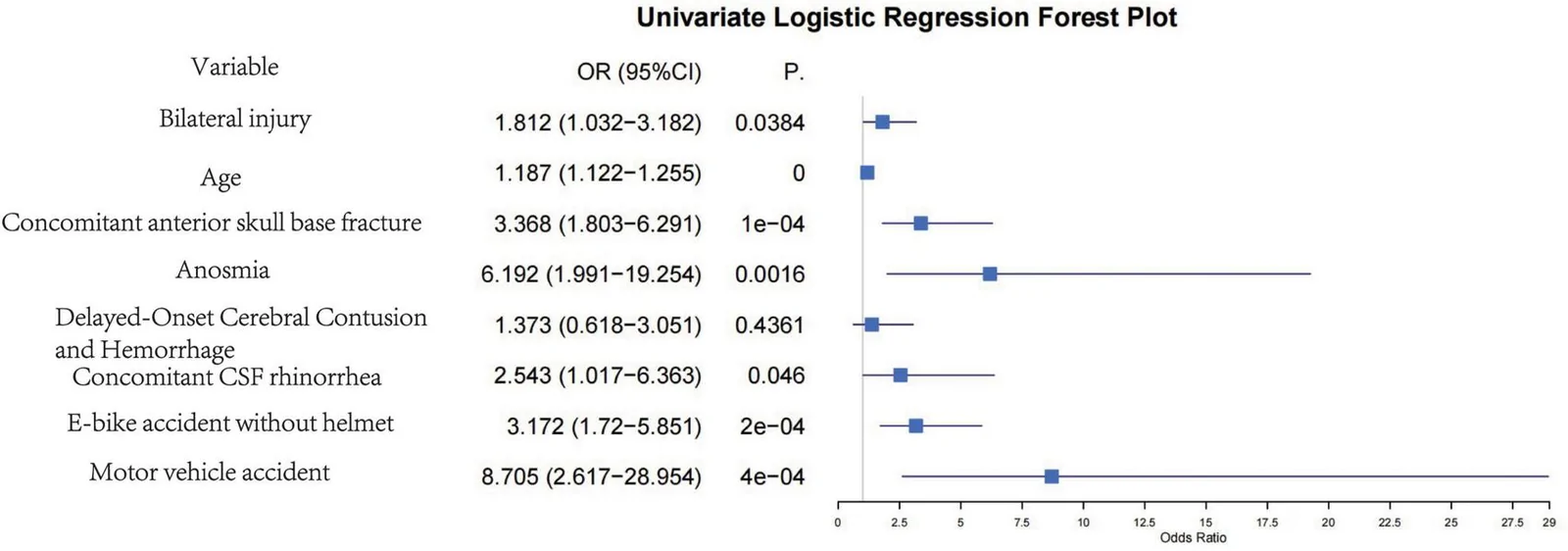
Univariate logistic regression forest plot of factors associated with cognitive impairment. OR, odds ratio; CI, confidence interval.

To further identify factors associated with cognitive impairment, univariate logistic regression analysis was performed. The results are presented in Table 3. Age was significantly associated with the occurrence of cognitive impairment (OR = 1.187, 95% CI: 1.122–1.255, *P* < 0.00001). Compared with fall-related injuries, both e-bike accident without helmet (OR = 3.172, *P* = 0.0002) and motor vehicle accidents (OR = 8.705, *P* = 0.0004) significantly increased the risk of cognitive impairment. Among clinical variables, contusion and hemorrhage volume (OR = 1.116, *P* = 0.0072) and the presence of anterior skull base fractures (OR = 3.368, *P* = 0.0001) were significantly associated with cognitive impairment. Furthermore, anosmia was strongly associated with an increased risk of cognitive impairment (OR = 6.192, *P* = 0.0016). The forest plot of the univariate analysis is shown in Figure 2.

**TABLE 3:** Univariate logistic regression analysis of factors associated with cognitive impairment.

Variable	OR	95% CI	*P*-value
Demographic characteristics			
Age, years	1.187	(1.122, 1.255)	< 0.00001*
Sex			
Reference group: Male	–	–	–
Female	0.888	(0.512, 1.542)	0.6731
Mechanism of injury, n (%)			
Reference group: Fall	–	–	–
E-bike accident with helmet	15422713.020	–	0.9843
E-bike accident without helmet	3.172	(1.720, 5.851)	0.0002*
Motor vehicle accident	8.705	(2.617, 28.954)	0.0004*
Clinical characteristics			
Admission GCS (points)	0.987	(0.871, 1.119)	0.8372
Severity of traumatic brain injury, n (%)			
Reference group: mTBI	–	–	–
Moderate TBI	1.054	(0.607, 1.830)	0.8525
Injury laterality, n (%)			
Reference group: Unilateral	–	–	–
Bilateral	1.812	(1.032, 3.182)	0.0384*
Frontal lobe cerebral contusion and hemorrhage volume	1.116	(1.030, 1.208)	0.0072*
Concomitant anterior skull base fracture, n (%)	3.368	(1.803, 6.291)	0.0001*
Concomitant EDH, n (%)	1.600	(0.765, 3.350)	0.2122
Concomitant SDH and SAH, n (%)	1.387	(0.791, 2.430)	0.2537
Concomitant CSF rhinorrhea, n (%)	2.543	(1.017, 6.363)	0.0460*
History of hypertension, n (%)	1.500	(0.832, 2.704)	0.1774
History of ICH, n (%)	1.054	(0.275, 4.045)	0.9388
History of ischemic stroke, n (%)	0.984	(0.440, 2.201)	0.9682
History of CAD, n (%)	1.079	(0.531, 2.193)	0.8327
Anosmia, n (%)	6.192	(1.991, 19.254)	0.0016*
Duration of mannitol therapy	1.037	(0.952, 1.130)	0.4034
Average daily dose of mannitol	1.002	(0.997, 1.006)	0.4716
Delayed-onset cerebral contusion and hemorrhage, n (%)	1.373	(0.618, 3.051)	0.4361
LOS	1.037	(0.972, 1.106)	0.2711

CAD, coronary artery disease; CSF, Cerebrospinal Fluid; EDH, Epidural Hematoma; ICH, intracerebral hemorrhage; SDH, Subdural Hematoma; SAH, Subarachnoid Hemorrhage; mTBI, mild Traumatic Brain Injury; GCS, Glasgow Coma Scale; OR, odds ratio; CI, confidence interval; LOS, Length of Hospital Stay.

*P < 0.05 indicates statistical significance. Variables with *P* < 0.1 in univariate analysis were considered candidates for multivariate logistic regression. –, Confidence interval could not be estimated.

### Multivariate firth logistic regression analysis

**FIGURE 3:**
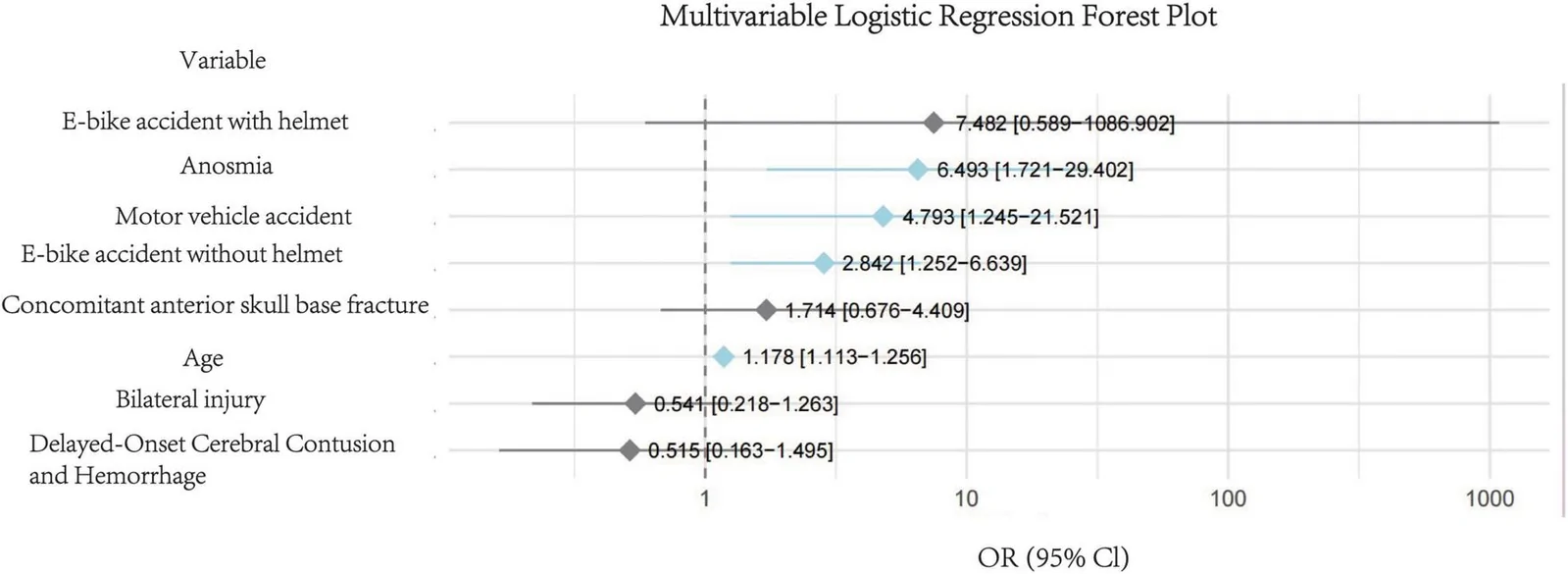
Multivariable Firth logistic regression forest plot of factors associated with cognitive impairment. OR, odds ratio; CI, confidence interval.

Variables with *P* < 0.1 in the univariate analysis were included in the Firth logistic regression model for multivariate analysis. The results are presented in Table 4. Age remained an independent risk factor for cognitive impairment (OR = 1.178, 95% CI: 1.113–1.255, *P* < 0.00001). Compared with fall-related injuries, electric scooter accidents without helmet use (OR = 2.842, *P* = 0.0124) and motor vehicle accidents (OR = 4.793, *P* = 0.0219) remained significantly associated with an increased risk of cognitive impairment. In addition, anosmia was identified as an independent risk factor (*P* = 0.0049). However, injury laterality, delayed cerebral contusion/hemorrhage, and anterior skull base fracture were not statistically significant in the multivariate model. The forest plot of the multivariate analysis is shown in Figure 3.

**TABLE 4:** Multivariable Firth logistic regression analysis of factors associated with cognitive impairment.

Variable	OR	95% CI	*P*-value
Demographic characteristics			
Age, years	1.178	(1.113, 1.255)	< 0.00001*
Mechanism of injury, n (%)			
Reference group: Fall	–	–	–
E-bike accident with helmet	7.482	(0.589, 1086.902)	0.1330
E-bike accident without helmet	2.842	(1.252, 6.639)	0.0124*
Motor vehicle accident	4.793	(1.245, 21.521)	0.0219*
Clinical characteristics			
Injury laterality, n (%)			
Reference group: unilateral	–	–	–
Bilateral	0.541	(0.218, 1.263)	0.1581
Anosmia, n (%)	0.987	(0.871, 1.119)	0.0049*
Delayed-onset cerebral contusion and hemorrhage, n (%)	0.515	(0.163, 1.495)	0.2259
Concomitant anterior skull base fracture, n (%)	1.714	(1.113, 1.256)	0.2556

OR, odds ratio; CI, confidence interval;

*P < 0.05 indicates statistical significance. Variables with P < 0.1 in univariate analysis were considered candidates for multivariate logistic regression. –, Confidence interval could not be estimated.

### Performance of the predictive model

#### Model discrimination

**FIGURE 4:**
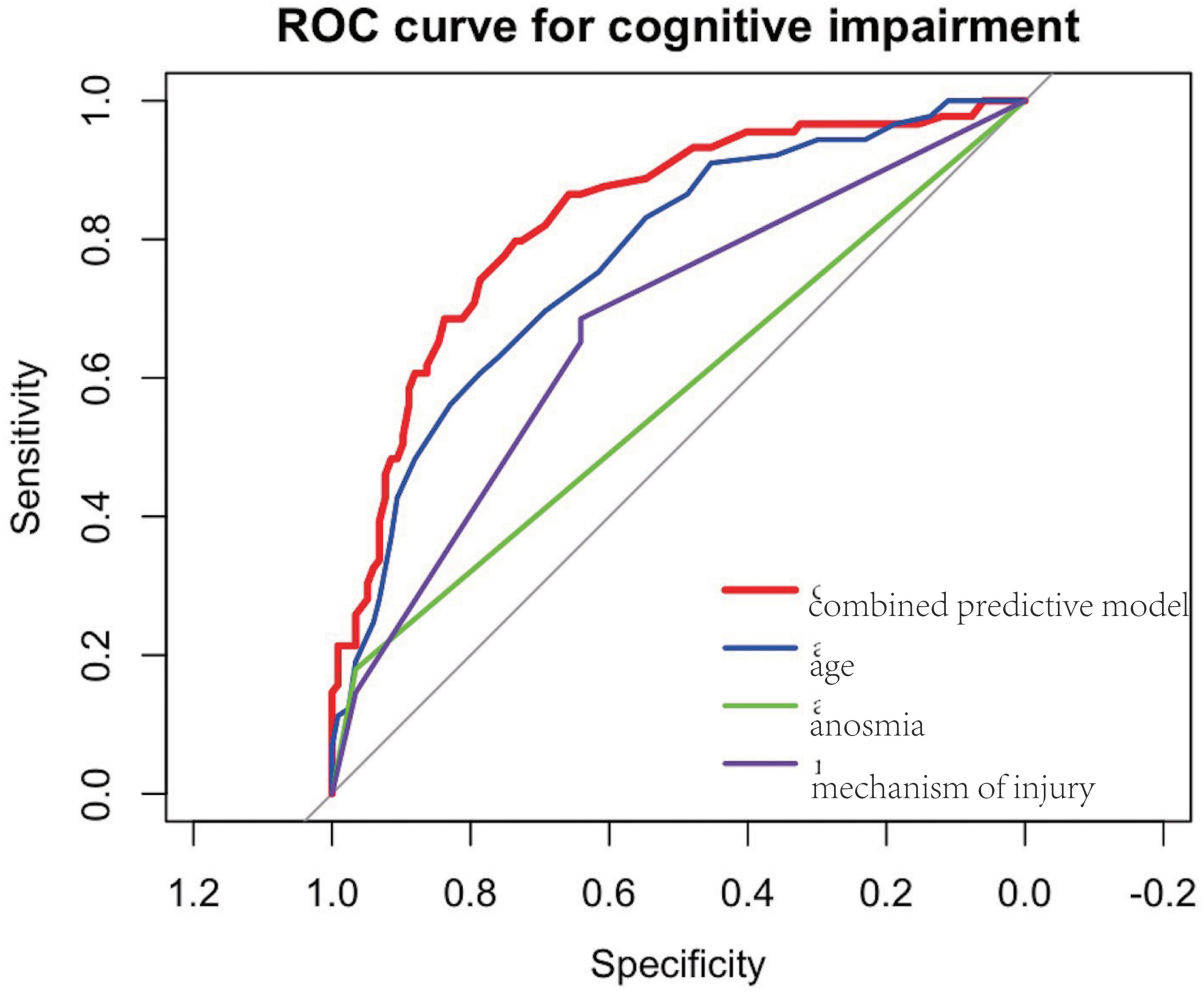
Receiver operating characteristic (ROC) curves for predicting cognitive impairment. The red curve represents the combined predictive model (AUC - 0.83), the blue curve represents age (AUC = 0.77), the green curve represents anosmia detected during treatment (AUC = 0.573), and the purple curve represents mechanism of injury! AUC = 0.672).

The ROC curve was used to evaluate the discriminative ability of the predictive model. The combined prediction model achieved an AUC of 0.83, indicating good discriminative performance. In comparison, the AUC values of individual predictors were: Age: 0.77; Mechanism of injury: 0.672; 0.573 for anosmia. The ROC analysis results for the individual predictors are summarized in Table 5. Numerically, the combined model showed a higher AUC than the individual predictors (Figure 4). However, no formal statistical comparisons were performed.

**TABLE 5:** Receiver-operating curve analysis results.

Variable	AUC(95%CI)	*P*-value
Demographic characteristics		
Age, years	0.77(1.126–1.270)	< 0.0001
Mechanism of injury, n (%)	0.672(1.184–2.209)	0.0027
Clinical characteristics		
Anosmia, n (%)	0.537(1.702–25.461)	0.0085

AUC, area under the curve; CI, confidence interval.

#### Model calibration

**FIGURE 5:**
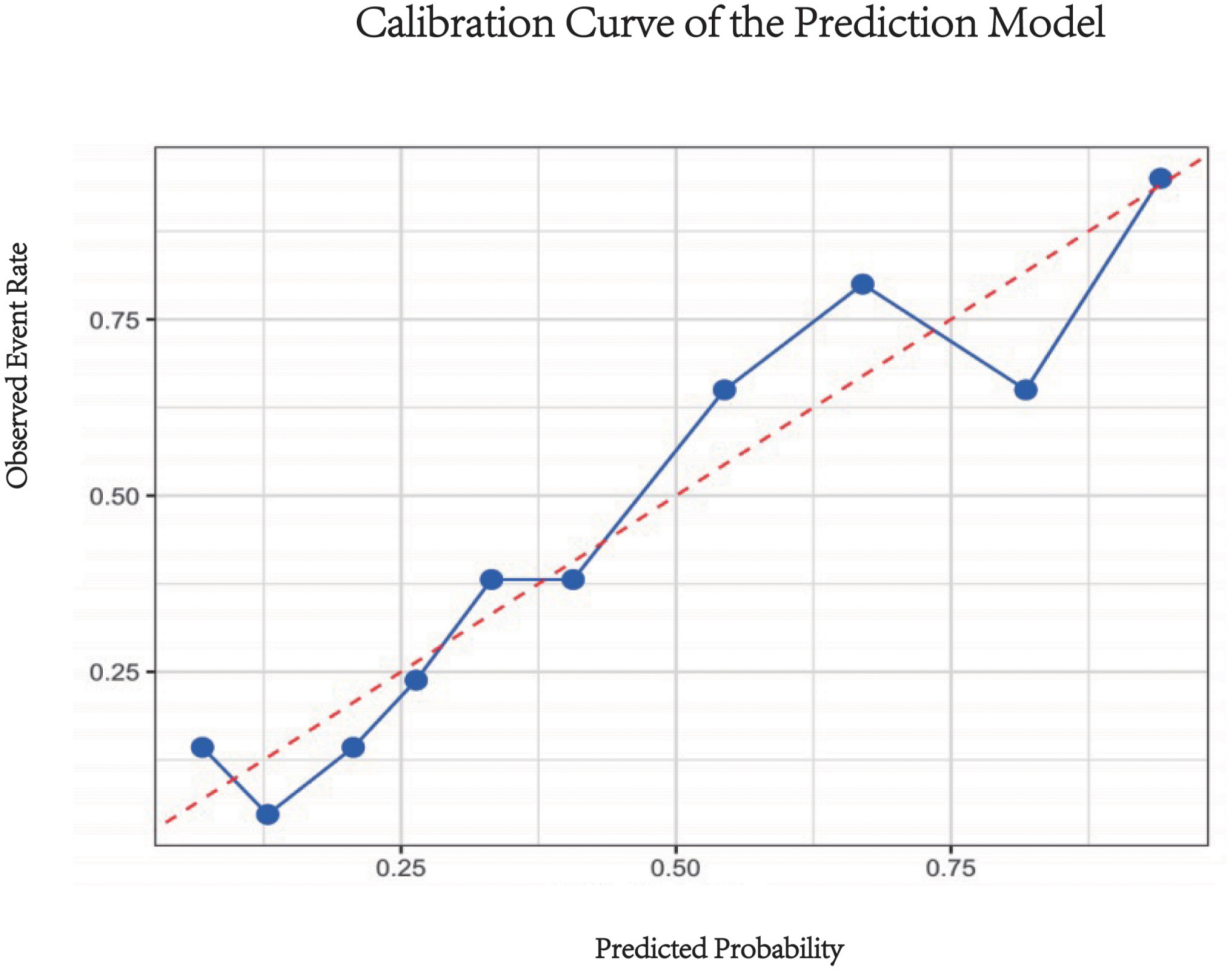
Calibration curve of the prediction model. The x-axis represents the predicted probability and the y-axis represents the observed event rate. The dashed red line indicates perfect calibration, while the blue line represents the model’s calibration performance.

Model calibration was assessed using the Hosmer–Lemeshow goodness-of-fit test and a calibration curve (Figure 5). The predicted probabilities were generally consistent with the observed event rates, and the calibration curve was close to the ideal reference line. Good agreement between predicted and observed values was observed across most risk ranges, particularly within the moderate probability range (approximately 0.3–0.7). Although a slight deviation was observed at higher predicted probabilities (approximately 0.7–0.8), the overall calibration performance of the model remained satisfactory.

#### Clinical utility

**FIGURE 6:**
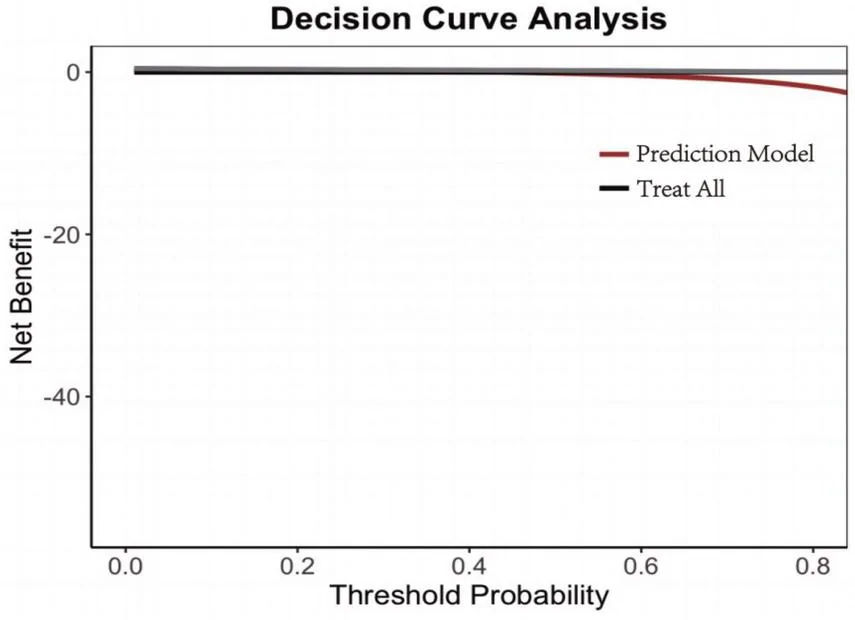
Decision curve analysis of the prediction model for cognitive impairment. The x-axis represents the threshold probability and the y-axis represents the net benefit. The red line represents the prediction model, the black line represents the treat-all strategy, and the gray line represents the treat-none strategy.

The clinical utility of the predictive model was evaluated using DCA (Figure 6). The results showed that, within a certain range of threshold probabilities, the predictive model provided a higher net benefit than both the “treat-all” and “treat-none” strategies. This suggests that the model may have potential clinical value in identifying patients at high risk of cognitive impairment.

## Discussion

The frontal lobe plays a critical role in cognitive function, and accumulating evidence indicates that cognitive impairment in various neurological disorders—including TBI, Alzheimer’s disease, and frontotemporal dementia—is closely associated with structural and functional damage to the frontal cortex. Previous studies have shown that the severity of cognitive impairment in patients with Alzheimer’s disease is associated with altered functional connectivity within the left frontal cortex (Franzmeier et al., 2018). Higher educational attainment may enhance cognitive reserve(CR) in the frontal cortex and mitigate cognitive decline during the prodromal stage of Alzheimer’s disease (Nelson et al., 2021; Zhu et al., 2021). However, these observations should not be interpreted as indicating that TBI and Alzheimer’s disease share equivalent pathogenic mechanisms. TBI is initiated by an acute mechanical insult followed by secondary processes such as neuroinflammation, blood–brain barrier disruption, cerebral edema, vascular injury, and axonal damage, whereas Alzheimer’s disease is a chronic progressive neurodegenerative disorder characterized by amyloid-β and tau pathology, synaptic dysfunction, and neuronal loss.

In addition, a history of TBI has been associated with long-term gray matter loss and atrophy in the orbital and medial frontal regions, which may be related to persistent cognitive impairment and subsequent post-traumatic neurodegenerative processes and increase the risk of mild cognitive impairment (MCI) (Khoury et al., 2024).

Frontal lobe cerebral contusion can lead to structural and functional damage to the frontal cortex, representing an important pathological basis for cognitive impairment following TBI. However, the occurrence and severity of cognitive dysfunction are not solely determined by frontal lobe injury itself but are also influenced by multiple injury-related factors. Moreover, post-traumatic cognitive impairment should be interpreted within a network-based framework rather than being attributed solely to focal frontal tissue damage. Executive function, attention, working memory, and other cognitive processes depend on distributed frontal-subcortical and frontal-limbic circuits involving interconnected cortical and subcortical regions. Therefore, cognitive impairment following frontal lobe contusion may reflect both focal cortical injury and disruption of broader structural and functional networks. In the present study, patients in the cognitive impairment group exhibited persistently lower cognitive performance throughout the follow-up period. Although cognitive function gradually improved between 1 and 6 months after injury, the overall cognitive performance remained significantly lower than that of patients without cognitive impairment. These findings suggest that cognitive recovery following frontal lobe contusion is a dynamic process and that clinically relevant impairment may persist despite partial improvement.

In this study, several factors—including age, mechanism of injury, contusion and hemorrhage volume, anosmia, and anterior skull base fracture—were closely associated with cognitive decline after TBI. Age, mechanism of injury, and anosmia were retained in the prediction model, whereas contusion and hemorrhage volume and anterior skull base fracture differed between the cognitive impairment and non-cognitive impairment groups in the initial analyses. These findings should be interpreted as clinical associations rather than evidence of causal biological pathways. These variables may reflect different aspects of injury severity, anatomical involvement, and individual susceptibility to post-traumatic cognitive impairment.

During TBI, the severity of primary and secondary brain injuries is closely related to neurological outcomes (Begemann et al., 2020). Our findings demonstrated that among patients with mild to moderate TBI, the volume of cerebral contusion and hemorrhage was significantly greater in the cognitive impairment group than in the non–cognitive impairment group. These results suggest that a greater initial lesion burden may be associated with poorer cognitive outcomes following frontal lobe TBI. More severe primary brain injury is often accompanied by extensive cortical disruption, axonal fiber damage, and cerebrovascular injury. These pathological changes may further trigger neuroinflammatory responses, cerebral edema, and metabolic disturbances, thereby exacerbating secondary brain injury and disrupting neural network integrity, potentially limiting cognitive recovery (Maas et al., 2022). Nevertheless, because contusion and hemorrhage volume was not retained as an independent predictor in the final model, its association with cognitive impairment may partly reflect overall injury severity or its relationship with other clinical variables.

Regarding the mechanism of injury, the present study found that patients injured in electric scooter accidents without helmet use and motor vehicle collisions exhibited more severe cognitive impairment than those injured by falls. This finding may be related to the biomechanical characteristics of different injury mechanisms. Traffic accidents typically involve higher impact energy and greater rotational acceleration, which are more likely to cause diffuse axonal injury (DAI) and structural damage to the frontal cortex (Johnson et al., 2013). As the frontal lobe is a critical center for higher cognitive functions—including executive function, attention, and working memory—structural damage to this region may be associated with cognitive decline. Furthermore, protective devices such as helmets may reduce the direct impact forces transmitted to the cranial structures. However, detailed biomechanical indicators, including collision speed, direction of impact, and rotational force, were not available in this retrospective study. Therefore, the observed association cannot be attributed exclusively to helmet use or to a specific biomechanical mechanism. Nevertheless, these findings support the general clinical importance of head protection and injury-prevention measures.

Anosmia is one of the common neurological deficits following TBI. In this study, the incidence of long-term anosmia was significantly higher in patients with cognitive impairment than in those without cognitive impairment, suggesting that anosmia may be closely associated with post-traumatic cognitive decline. The most plausible explanation for this association is that anosmia reflects the severity and anatomical location of the primary injury, particularly involvement of the olfactory nerve fibers, olfactory bulb or tract, orbitofrontal cortex, and anterior skull base. Because these structures are anatomically and functionally connected with frontal-limbic networks involved in executive function, memory, emotion, and behavior, injury to these adjacent systems may result in the concurrent occurrence of olfactory and cognitive dysfunction. Accordingly, anosmia may serve as a clinically observable marker of a broader anterior cranial injury pattern rather than being a direct cause of cognitive impairment. Recent studies have shown that compared with TBI patients without olfactory impairment, those with olfactory loss may exhibit significant impairments in neurobehavioral domains such as feeding behavior, sleep regulation, and motor activity, although differences in certain cognitive domains such as visual perception, attention, and memory may be less pronounced (Langdon et al., 2021). Therefore, the association observed in the present study may reflect shared anatomical injury or greater overall injury burden, and the relationships among anosmia, injury location, and specific cognitive domains require further investigation.

Moreover, anosmia is also considered an early clinical manifestation of neurodegenerative diseases such as Alzheimer’s disease. However, comparisons between olfactory dysfunction in Alzheimer’s disease and TBI should be interpreted cautiously because their temporal courses and underlying pathophysiological processes differ substantially. In Alzheimer’s disease, olfactory dysfunction develops in the context of progressive neurodegeneration and amyloid-β and tau pathology. In contrast, post-traumatic anosmia may result from acute shearing of the olfactory nerve fibers, injury to the olfactory bulb or tract, contusion of the orbitofrontal cortex, or anterior skull base fracture. Accordingly, the occurrence of olfactory dysfunction in both conditions does not establish a shared disease mechanism.

Although experimental studies suggest that lymphatic pathways in the nasal cavity, cribriform plate region, and skull base may participate in cerebrospinal fluid drainage and the clearance of brain-derived solutes (Phillips and Schwartz, 2024), the present study provides no direct evidence that anosmia reflects meningeal lymphatic dysfunction. Meningeal lymphatic anatomy and drainage function were not assessed, and anosmia cannot be considered a surrogate marker of impaired meningeal lymphatic drainage on the basis of the present findings. Therefore, the possible relationship among olfactory pathway injury, meningeal lymphatic dysfunction, and cognitive impairment remains hypothetical and secondary to the more direct explanation involving the severity and anatomical location of the primary injury.

Another noteworthy finding in this study was that the incidence of anterior skull base fractures was significantly higher in patients with cognitive impairment. As a common concomitant injury in frontal lobe impact trauma, anterior skull base fractures are often associated with dural contusion or laceration, and severe cases may lead to complications such as CSF rhinorrhea or otorrhea. Such fractures may coexist with injury to the dura mater, olfactory nerve fibers, frontal cortex, and adjacent vascular structures. Consequently, anterior skull base fracture may serve as an indicator of greater local injury burden. The coexistence of anosmia and anterior skull base fracture may therefore primarily reflect a shared pattern of anterior cranial injury involving the olfactory pathways and adjacent frontal regions. It is also biologically conceivable that trauma involving the skull base could affect adjacent meningeal lymphatic structures; however, this possibility was not directly evaluated in the present study and should not be regarded as the primary explanation for the observed association between anosmia and cognitive impairment.

Recent studies have demonstrated that meningeal lymphatic vessels (mLVs) located at the skull base possess unique structural features, including button-like endothelial junctions and lymphatic valves, which facilitate fluid absorption and drainage (Louveau et al., 2015). In addition, skull base mLVs are located adjacent to the subarachnoid space, suggesting their possible involvement in the clearance of macromolecules from the cerebrospinal fluid (Ahn et al., 2019). Nevertheless, whether anterior skull base fractures disrupt these lymphatic structures or impair cerebrospinal fluid clearance in patients with TBI remains unknown. Furthermore, the frontal lobe—an essential region for cognitive processing—is anatomically located above the irregular bony ridges of the anterior skull base. When anterior skull base fractures occur, relative movement or collision between the frontal lobe and these bony ridges may aggravate cerebral contusion, thereby causing direct damage to cognition-related neurons and neural fiber pathways. Thus, the observed association between anterior skull base fracture and cognitive impairment may reflect direct frontal injury, olfactory pathway damage, disruption of broader neural networks, or other secondary injury processes. The potential involvement of meningeal lymphatic dysfunction should be regarded as a hypothesis requiring direct investigation rather than an established mechanism.

Future studies incorporating dedicated imaging of meningeal lymphatic drainage, cerebrospinal fluid or blood biomarkers, and comprehensive neuropsychological assessments may help determine whether alterations in this system are associated with cognitive outcomes after TBI. Such investigations would also be necessary to distinguish the potential effects of lymphatic dysfunction from those of direct frontal injury, olfactory pathway damage, diffuse network disruption, neuroinflammation, and other secondary pathological processes.

In summary, this study systematically analyzed the clinical characteristics and injury-related factors in patients with mild to moderate frontal lobe TBI. Age, injury mechanism, and anosmia were identified as clinically relevant predictors of cognitive impairment in patients with predominant frontal lobe cerebral contusion. Contusion and hemorrhage volume and anterior skull base fracture also differed between the cognitive outcome groups in the initial analyses but were not retained as independent predictors in the final model. Cognitive impairment in this population may reflect the combined influence of focal frontal injury and disruption of distributed neural networks. Anosmia may primarily serve as a clinical marker of the severity and anatomical location of the primary injury, particularly involvement of the olfactory pathways, orbitofrontal cortex, anterior skull base, and interconnected frontal-limbic networks Although skull base meningeal lymphatic dysfunction represents a potentially relevant direction for future research, it was not directly evaluated in the present study, and anosmia should not be interpreted as evidence of impaired meningeal lymphatic drainage. Accordingly, and no mechanistic or causal conclusions regarding its role can be drawn from the current findings.

## Limitations

Although this study provides clinical evidence for exploring the mechanisms and potential therapeutic strategies for cognitive impairment following TBI, several limitations should be acknowledged. First, this was a single-center retrospective study, which may introduce selection bias. Second, the sample size was relatively limited, and future multicenter studies with larger cohorts are required for external validation and for further investigation of the role of the skull base meningeal lymphatic system in the development of post-traumatic cognitive impairment. In addition, detailed neuroimaging indicators and comprehensive neuropsychological assessments were not included in this study, which might further improve the predictive accuracy of the model.

Finally, the restriction of the study population to patients with predominant frontal lobe cerebral contusion should also be considered when interpreting the findings. Although this restriction reduced anatomical heterogeneity and limited the potential confounding effects of injuries predominantly involving other brain regions, post-traumatic cognitive impairment is increasingly recognized as a consequence of disruption across distributed neural networks rather than damage to a single anatomical region. Consequently, the present findings may not be directly generalizable to patients with diffuse axonal injury, predominant non-frontal injury, or more heterogeneous patterns of TBI. Future studies involving diverse injury locations and patterns are warranted to evaluate the broader applicability of the prediction model.

## Conclusion

The incidence of cognitive impairment in patients with frontal lobe cerebral contusion is relatively high. Age, mechanism of injury, and anosmia were identified as important independent predictors of cognitive impairment. The predictive model constructed based on these factors demonstrated good discrimination, acceptable calibration, and potential clinical utility, and may provide a useful tool for early risk assessment of cognitive impairment in patients with frontal lobe cerebral contusion.

## Data Availability

The raw data supporting the conclusions of this article will be made available by the authors, without undue reservation.
